# Skeletal, Dentoalveolar and Dental Changes after “Mini-Screw Assisted Rapid Palatal Expansion” Evaluated with Cone Beam Computed Tomography

**DOI:** 10.3390/jcm11164652

**Published:** 2022-08-09

**Authors:** Patricia Solano Mendoza, Paula Aceytuno Poch, Enrique Solano Reina, Beatriz Solano Mendoza

**Affiliations:** Department of Orthodontics and Dentofacial Orthopedics, School of Dentistry, University of Seville, 41009 Sevilla, Spain

**Keywords:** micro implant-assisted rapid palatal expansion, maxillary transverse deficiency, Cone-beam computed tomography, alveolar bone, midpalatal suture, skeletal expansion, palatal expansion

## Abstract

The purpose of this study was to evaluate skeletal, dentoalveolar and dental changes after Mini-screw Assisted Rapid Palatal Expansion (MARPE) using tooth bone-borne expanders in adolescent patients after analyzing different craniofacial references by Cone beam computed tomography (CBCT) and digital model analysis. This prospective, non-controlled intervention study was conducted on fifteen subjects (mean age 17 ± 4 years) with transversal maxillary deficiency. Pre (T1) and post-expansion (T2) CBCTs and casts were taken to evaluate changes at the premolars and first molar areas. To compare means between two times, paired samples *t-* or Wilcoxon test were used following criteria. Significant *skeletal changes* were found after treatment for Nasal width and Maxillary width with means of 2.1 (1.1) mm and 2.5 (1.6) mm (*p* < 0.00005). Midpalatal suture showed a tendency of parallel suture opening in the axial and coronal view. For *dentoalveolar* changes, a significant but small buccal bone thickness (BBT) reduction was observed in all teeth with a mean reduction of 0.3 mm for the right and left sides, especially for the distobuccal root of the first molar on the left side (DBBTL1M) [IC95%: (−0.6; −0.2); *p* = 0.001] with 0.4 (0.4) mm. However, a significant augmentation was observed for the palatal bone thickness (PBT) on the left side. The buccal alveolar crest (BACL) and dental inclination (DI) showed no significant changes after treatment in all the evaluated teeth. MARPE using tooth bone-borne appliances can achieve successful skeletal transverse maxillary expansion in adolescent patients, observing small dentoalveolar changes as buccal bone thickness (BBT) reduction, which was not clinically detectable. Most maxillary expansions derived from skeletal expansion, keeping the alveolar bone almost intact with minor buccal dental tipping.

## 1. Introduction

Transversal maxillary deficiency (TMD) is a quite common condition that affects between 8–23% of deciduous and mixed dentitions. However, a lower prevalence has been reported in adult orthodontic patients [1,2,3,4]. This transverse deficiency [5], or maxillary hypoplasia [6], is one of the main problems related to facial growth that should be corrected as it is diagnosed, with the objective to reestablish a normal transverse skeletal relationship between maxillary and mandibular basal bones to obtain a stable occlusion [7].

Its etiology is multifactorial, frequently influenced by myofunctional disorders of the stomatognathic system and generally associated with oral breathing or deleterious habits such as thumb sucking [4,8,9]. Genetic and hereditary factors are also related, thus determining the development of maxillary transverse deficiencies. These factors promote structural changes in the maxilla which will generally lead to posterior crossbite (bilateral or unilateral), constriction of the nasal cavity and frequent dental crowding [8,10].

Traditionally, orthopedic rapid maxillary expansion (RME) has been performed to correct this matter during patient’s growing period showing positive results with a better prognosis and treatment outcomes at early ages [5,11,12,13,14], producing a greater orthopedic effect in the deciduous and mixed dentition. As the patient grows, progressive calcification, and craniofacial sutures interdigitation occur, including midpalatal suture closure. Consequently, skeletal expansion becomes a more difficult process due to increased mechanical resistance [15,16]. Limited skeletal orthopedic changes have been described after RME with the use of tooth-borne expanders observing undesirable side effects such as dental tipping, buccal bone thickness reduction, bone dehiscence and gingival recession in anchor teeth [17,18,19,20,21,22], limiting this treatment option to incomplete skeletal mature patients, confirming the importance of early skeletal age treatment [23].

Skeletal support with the use of screws by means of mini-screw assisted rapid palatal expansion (MARPE) allows for a better distribution of applied forces when the palatal suture closure is incomplete, thus achieving a greater skeletal effect (orthopedic) by opening the mid-palatal suture and minimizing secondary effects derived from dentoalveolar (orthodontic) effects [24]. Cone-beam computed tomography (CBCT) images have revealed a significant increase in the skeletal dimension in adolescent and young adult patients treated with MARPE [25], reducing the aforementioned side effects when the intermaxillary suture is not completely closed [26,27,28,29], and, therefore, considering MARPE as a proper treatment option for correcting maxillary transversal discrepancies (MTD) [30,31,32] with potential skeletal effect [33,34]. Studies show conflicting results regarding the orthopedic effect MARPE and RME in young adult patients with different types of appliances [35].

Specifically, tooth bone-borne expanders with palatal minis crews and first molar anchorage have shown effective and positive results for maxillary expansion [33,36,37], with no risk of periodontal damage.

As studies using computed tomography (CT) report, buccal alveolar bone thinning after RME in anchored teeth [20,38], the use of CBCT seems to be fundamental, contributing to a more complete diagnosis of the transverse dimension [29,39] providing accurate tridimensional treatment outcomes evaluations of the maxillofacial complex and their dentoalveolar response.

This was the main reason that led us to hypothesize that MARPE using tooth bone-borne expanders could be a safe and effective treatment to correct maxillary transversal discrepancies in adolescent patients with incomplete ossification of the maxillary palatal suture achieving higher predictable skeletal effects with reduced dentoalveolar and dental effects.

The purpose of this study is to evaluate skeletal, dentoalveolar and dental changes after MARPE using tooth bone-borne expanders in patients with incomplete maxillary palatal suture closure analyzing different craniofacial references by CBCT and digital model analysis in order to quantify maxillary transverse changes at the three levels after expansion treatment. Moreover, we tried to show the quantity of skeletal, alveolar and dental responses to treatment and compare these findings with those that are reported in the literature.

## 2. Material and Methods

This prospective, non-controlled intervention study was conducted on patients who needed maxillary expansion. Patients were recruited from three different centers in the same city, Seville (Spain): The Department of Orthodontics of the School Dentistry of the University of Seville, a private dental clinic (COINSOL) and a dental training institute IDEO. All subjects met the following inclusion criteria: (1) presence of transverse skeletal maxillary deficiency with or without the presence of posterior crossbite as described by Tamburino et al. [40,41], (2) incomplete radiographic ossification of the midpalatal suture according to Angelieri’s classification [42], (3) not having received previous orthodontic treatment, (4) presence of first and seconds upper premolars and first upper molars, (5) absence of any craniofacial irregularities, (6) and any bone defects, or systemic and periodontal disease, (7) not being pregnant (8) and having reached prepubertal development with less than 25 years of age. Palatal suture ossification was evaluated following this classification, categorizing the palatal maturation in five stages (A–E) through its CBCT analysis in an axial view. Patients who did not meet the inclusion criteria and those with a portion of the suture where the fusion had occurred (stage E in accordance with Angelieri’s classification) and with no low-density spaces along the suture were excluded. Incomplete ossification of mid palatal suture was blindly evaluated in the initial CBCT (CbctT1) to confirm that the patient could be included in the study.

All subjects gave their informed consent before taking part in the study. This study was conducted in accordance with the Declaration of Helsinki, and the protocol was approved by the Ethics Committee of Vírgen Macarena-Virgen del Rocío University Hospitals in Seville (238e1b02c492fe6c37e2e9cf37c737297f4cf746).

An initial sample of 19 patients meeting the established inclusion criteria were selected. Four patients were not included in the analysis: two patients due to incomplete radiographic registers, one patient used other orthodontic appliance at the same time and one patient had a different screw positioning. The final sample consisted of 15 patients between 13 and 24 years of age (mean age: 17.0 ± 4.0), with transverse maxillary skeletal compression (mean:5.4 ± 2.1 mm) who completed maxillary expansion (Table 1).

### 2.1. Methodology

**Figure 1:**
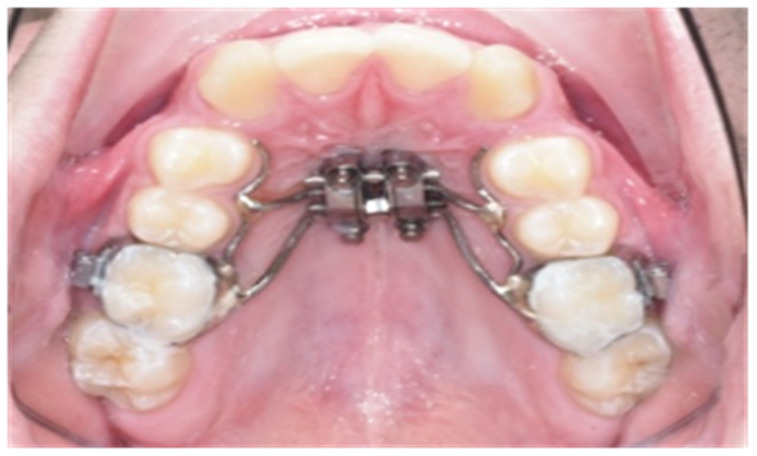
Tooth-bone-borne expander appliance.

Patients were treated by MARPE with a tooth-bone born expander using the MSE (maxillary skeletal expander designed by Dr. Moon from the University of UCLA) adapted to individual maxillary expansion requirements [43,44]. Four stainless steel arms of 1.5 mm in diameter emerged from a central main screw: two anterior arms extended symmetrically towards the palatal aspect of the first upper premolars near the most cervical part of the tooth (without support), and two posterior arms extended the palatal aspect of the first molars, which remained fixed to the bands placed on the first molars. A 0.7 mm thick steel lateral arm was connected to the expander’s front arms (Figure 1).

Every device was adapted individually for each patient in the same laboratory through an initial impression with the bands positioned on the upper first molars.

The central screw expander had four holes of 1.8 mm in diameter for the retention and insertion of each mini screw of 1.74 mm in diameter and 9, 11 and 13 mm in length which were used for bone anchoring. Anterior and posterior mini-screws were positioned at the level the first premolar and the first molar, respectively (BMK micro-screws model ACR of Medical Resources). To ensure a bicortical anchorage, palatal gingiva thickness, bone height and distance between the expander and palatal gingiva were considered [45]. The central screw expander length was selected based on the patient’s maxillary expansion needs to correct the transversal deficiency (8, 10 or 12 mm). The maxillary compression or maxillary transversal deficiency was calculated considering the difference between the mandibular and maxillary widths determined by the arch relationship according to Cantarella 2017 [44]. Expander placement and the first activation were performed in the same appointment, following the describe protocol suggested by Bruneto [8] according to patients age. The expander was activated by 1 turn per day (0.26 mm/turn) with subsequent biweekly visits until full expansion was achieved [8]. The total maxillary expansion was completed when the palatal cusp tips of the maxillary first molars were in contact with the corresponding buccal cusp tips of the mandibular first molars.

The same experienced examiner was present in the 3 centers (BSM) for the recruitment of patients enrolled in the study, confirming incomplete ossification of midpalatal suture and supervised successive visits until the expansion was completed, from February 2020 to December 2022 [46,47]. Radiographic CBCT (Cbct) and cast (Model) recordings were collected at two times: T1 (prior to expander placement) and T2 (immediately after maxillary expansion had been completed). The expander was removed before CbctT2 and ModelT2 to prevent image distortions, and immediately placed back without miniscrews, blocking it with ligature leaving the expansion device for a 6-month retention period. The patient’s skeletal maturation was assessed via the cervical vertebral maturation index (CVM) in the initial teleradiograph (CbctT1) in accordance with Bacetti’s classification and stratified in 6 stages from CVM I-VI [48,49] base of the presence or absence of concavity in the lower border of the body of C2, C3 and C4 and the body shape of C3 and C4.

Once the treatment was completed, all measurements were performed by two blinded, calibrated examiners. Radiographic measurements (PSM) and digital cast measurements (PAP) were performed, evaluating intraexaminer reproducibility. Skeletal and sutural maturation were assessed (PSM) from CbctT1 in accordance with the previously describe classifications [42,48,49]. Intraexaminer reproducibility was analyzed by using randomly selected patients. In order to assure a reliable reproducibility and blindness, each patient was assigned a unique code and measurements were recorded twice and spaced 1 week apart. 

Stone casts were digitally scanned with Itero^®^ Element 2 Scanner (Tel Aviv-Yafo, Israel) and analyzed for measurements with OrthoCAD^®^ software (OrthoCAD iCast Orthodontic 3D Digital Modeling Study of Align Technology, Inc., IL, USA) to evaluate changes at the following levels: first and second premolar and first molar (1PM, 2PM, 1M): (1) *Palatal Gingival Width* (PGW): distance from the palatal gingival margin of the tooth of interest to its contralateral, (2) *Palatal Cusp Width* (PCW): distance from the palatal cusp of the tooth of interest to its contralateral, (3) right and left *Clinical Coronal Height* (CCH) to evaluate gingival margin position changes: distance from the center of the gingival margin to the buccal cusp for the premolars and to the center of the crown in the buccal aspect for the first molar.

CBCT scan images were obtained with i-CAT^®^ Kavo 1723 flat panel model for IDEO and COINSOL patients, and with Planmeca Promax^®^ 3D Sirona (Finlandia, Helsinki) for Dental School patients. For i-CAT^®^ Kavo: 37 mA, 120 kV and 26 s scan time were set for T1 and T2. Voxel size of 0.2 mm. for both exposure times and a field of view of 16 × 13 cm FOV (cervical included). PlanMeca Promax 3D from Sirona system used same parameters for two evaluation periods T1 and T2, being: 14 mA, 90 KV, with a 12 s exposure time, 0.2 Vox and 8 × 8 FOV. Anatomage in Vivo 5,3. i-CAT^®^ Kavo software was used to perform all radiographic measurements with a 1:1 scale, slice thickness 0.5 mm. All CBCT volume images were reoriented prior to radiographic measurements considering three-dimensional reference planes for craniofacial structures orientation and to standardize linear measurements in the sagittal section (y-plan), axial section (x-plane) and coronal section (z-plan):

a. Radiographs orientation: In the axial section (x-plane), the mid-palatine suture was used. In the midsagittal section (y-plane), the horizontal palatal plane was the selected reference, considering the anterior and posterior nasal spine. In the coronal section (z-plane), the image was oriented perpendicular to the patient’s midsagittal plane tangent to the most inferior level of nasal floor [50].

b. Cuts standardization: Points were established in the coronal, axial and sagittal plane at selected teeth: first and second premolars and first molar (1 PM, 2 PM, 1 M) in line with previous validated cut Standardization and radiographic measurement method described by Podesser [50] on CT, and by Christie [51] and Toklu [29] on CBCT.

**Figure 2:**
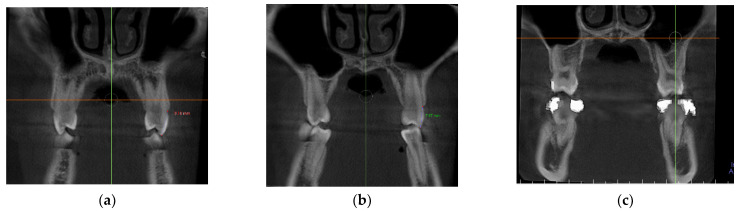
Selected cut-off points in the coronal plane at: (**a**) first premolar, (**b**) second premolar and (**c**) first molar.

**Figure 3:**
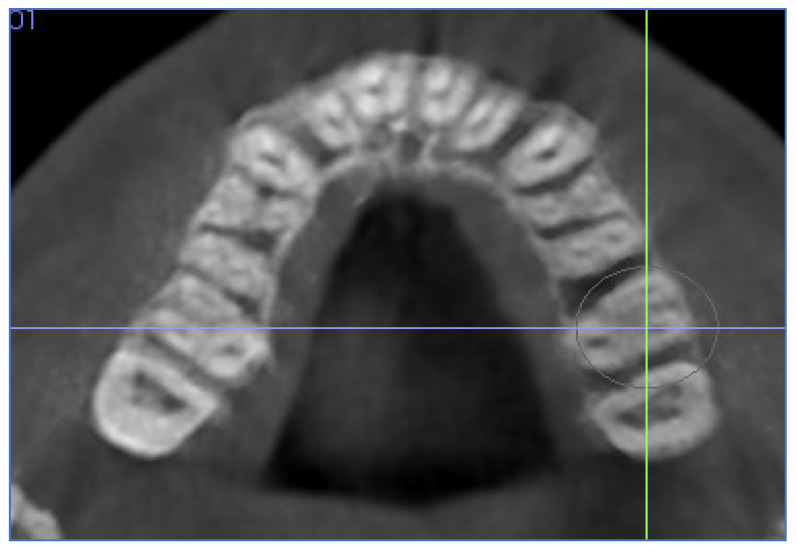
Cut in the axial plane for buccal and palatal bone thickness (BBT and PBT) measurements.

– In the coronal plane: For the first premolar and the first molar: cuts were made at the most anterior section where the crown and palatal root can be seen at their greatest length. For the second premolar: in the most anterior section showing maximum length of its root (Figure 2).

– In the axial plane: at the level of the right and left first molar trifurcation for each side (Figure 3). (Maxillary right first molar furcation for the right posterior teeth and the maxillary left first molar furcation for the left posterior teeth) According to Toklu [29].

Radiographic changes were evaluated at selected teeth for the following variables: (1) **Skeletal changes**: *Nasal width, maxillary width, palatal suture opening, sutural expansion, nasal floor, palatal floor*, (2) **Dentoalveolar changes**: *buccal maxillary width and palatal maxillary width*, on left (L) and right (R) side of the upper arch: *buccal bone thickness*, *palatal bone thickness* and *buccal alveolar bone crest Level*, (3) **Dental changes**: *dental inclination* (Table 2).

Midpalatal suture expansion was also assessed in the coronal view, measured in the middle of the palate as sutural expansion (SEM), and at the level of *nasal and palatal floor* on a coronal cross-sectional slice through the center of the first molar, by connecting the right and left external edges of the suture according to the previous method used by Ngan [34]. (Table 3). The suture external edges were verified in the axial cross-sectional slice for each tested position.

The skeletal expansion was calculated by the radiographic analysis of: *Sutural expansion* (SEM), *intermolar width* (IMW), and *palatal maxillary width* (PMW) in compliance with the method proposed by Ngan et al. 2018 [34] (Table 4).

Total expansion (TE) included the skeletal (separation of two maxillary halves at the midpalatal suture) and dentoalveolar expansion (alveolar bone bending and dental tipping). A mathematical equation was used to calculate the skeletal and dentoalveolar components of the Total expansion: TE = Skeletal (orthopedic) expansion: Midpalatal sutural separation + dentoalveolar (orthodontic) expansion (alveolar bone bending + dental tipping). Total expansion was defined as the change between the two time periods (T2–T1) in (1) *Intermolar width*, distance between the palatal cusp tip of the right and left first molars measured in a coronal cross-sectional slice through the midportion of the first molar. (2) *Sutural expansion* in the middle of the palate on the same coronal cross-sectional slice and (3) *palatal maxillary width*, measured at the first molar’s furcation on the same coronal cross-sectional slice (Table 4). From the dentoalveolar expansion: Alveolar bone bending, and dental inclination were also determined for each patient. Alveolar bone bending was defined as any additional palatal alveolar expansion achieved apart from the sutural separation and was calculated by subtracting *Sutural Expansion* (SEM) from the change (T2–T1) in *palatal maxillary width* (PMW). Dental inclination was computed by subtracting *sutural expansion* and the calculated alveolar bone bending from total expansion [34].

### 2.2. Statistical Analysis

The statistical analyses were performed with the Statistical Package, using SPSS 26.0 software for Windows (SPSS Inc. Chicago, IL, USA). For quantitative variables, those that presented a symmetric distribution, were presented as means and standard deviation (SD) and those that presented a very asymmetric distribution, as median and interquartile range (P_25_, P_75_) and frequency and percentages for the categorical variables. The 95% CIs have been calculated for all the statistics obtained. To assess changes after MARPE maxillary expansion at two evaluation moments, the *Student’s t*-test was performed for paired data once the randomness and normality requirements had been confirmed. In cases of not meeting the normality requirement (*Shapiro–Wilks test*), the non-parametric test (*Wilcoxon test*) was applied. To compare the means between two independent groups, the *Student’s t-test* was performed for independent data once the requirements of randomness, normality (*Shapiro Wilks test*) and equality of variance (*Levene’s t*) had been confirmed. If said requirements were not met, the *Student’s t-test* was performed for independent data with *Welch’s* correction. If the normality requirements were not met, a non-parametric test (*Mann–Whitney U*) was applied. Significance of the results was evaluated at the level of alpha < 0.05.

Based on the observed effect sizes, experimental statistical power analyses were conducted to determine the power of the study. The sample size was calculated to detect any clinically relevant differences in the reduction in buccal wall thickness of 0.5 mm after expansion [20,29]. Being the standard deviation of the differences 0.62, considering an alpha error of 0.05 and a power of 85%, the minimum number of subjects to be included in the study was 14 patients. ICCs assessment for intraexaminer reproducibility ranged between 0.85 and 0.98 for radiographic and cast measurement showing an important level of repeatability for all measurements.

## 3. Results

ICC were greater than 0.90 for most of the variables and greater than 0.83 for the second left premolar’s clinical crown height, the first premolar’s palatal maxillary width, the first premolar’s suture opening, the second right premolar’s buccal bone thickness, the first left molar’s buccal bone thickness and the second left premolar’s buccal bone thickness.

The initial maxillary transversal deficiency ranged from 2.5 to 8.7 mm with a mean of 5.4 (2.1) mm. The mean amount of screw expansion was 6.8 mm (1.8), ranging from 3.4 mm to 9.4 mm, with a mean of 22 (8.0) days, 95% CI: (17.0, 26.0). Sutural stage maturation evaluation according to Angelieri’s classification [42] confirming all sutures were not completely ossified before maxillary expansion: 86.7% [95% CI: (86.7; 93.3) of the sample (n = 13) presented midpalatal suture stages type C and 6.7% (n = 1) stage type B and D. Regarding skeletal maturation (CVM) according to Baccetti [48,49], most patients presented stages IV and V with a distribution of 46.7% (n = 7) and 40% (n = 6), respectively, and 13.3% stage III (Table 5).

### 3.1. Radiographic Measurements

CBCTs performed at the two evaluation times were needed to analyzed all evaluated parameters and existing changes after treatment at the skeletal, dentoalveolar and dental level.

*Skeletal changes*: The midpalatal suture was successfully opened showing a significant change in all patients. Suture opening (SO) in the axial view expressed a similar quantity at the premolar and molar areas, with a mean of 3.3 (1.3) mm for the first premolars, 2.9 (1.4) mm for the second premolars and 2.6 (1.3) mm for the first molars, showing a gradual opening tendency from the anterior to the posterior part with no statistically significant differences between teeth along the length of the midpalatal suture (Table 6).

Midpalatal suture expansion assessed on the coronal view showed a statistically significant change (*p* < 0.000) at the Nasal and Palatal Floor levels after maxillary expansion, not showing statistically significant differences between them.

*Dentoalveolar changes*: Statistically significant differences were found after maxillary expansion for buccal and palatal maxillary width in all analyzed teeth. Regarding buccal bone thickness (BBT), once the maxillary expansion was completed, all locations showed similar significant changes with a mean reduction of −0.3 mm for both sides (Table 7). Although these values show statistically significant differences, they are not clinically relevant. In the palatal aspect, bone thickness (PBT) only showed significant changes on the left side, on the first premolar and the molar, respectively. The buccal alveolar crest remained with hardly any changes, not being significant at any locations.

*Dental changes:* Dental inclination did not show significant variation after treatment being minimal on all the evaluated teeth. The reduction in the measured angle shows a tendency to positive tipping, slightly increasing from the first molar to the first premolar (Table 8).

### 3.2. Cast Measurements

Palatal gingival widths and palatal cusp widths showed significant changes in all the teeth after MARPE. Regarding the height of the clinical crown (CCH), significant minor changes were observed only on the left side, with a mean change of 0.2 (0.2) mm, 0.1 (0.2) mm and 0.1 (0.1) mm for premolars and the first molar, respectively. However, none of these small increases were clinically noticeable.

A total expansion of 4.5 (1.8) mm was obtained after MARPE, defined as the change in the IMW for the first molar. Greater expansion amount (2.7 (1.0) mm) corresponds to skeletal component and a lower proportion (1.8 (1.7) mm) to dentoalveolar expansion, both changes being statistically significant. Significant changes were observed for IMW, PMW and SEM. From the dentoalveolar expansion, the alveolar bone bending effect and the dental inclination showed reduced mean values of 0.7 (1.6) mm and 1.1 (1.3) mm, respectively. The amount of skeletal expansion achieved within the total expansion was 60 %, determined by mid-palatal suture expansion [2.7 (1.0) mm] in the center of the palate at the first’s molar level, meaning 40% remaining corresponds to the dentoalveolar component (1.8 mm). Flexion of the alveolar bone accounted for 15,5% of TE. The remaining fraction of TE in the first molar resulted from dental inclination was 24.4%.

## 4. Discussion

It is generally accepted that chronological age is not a precise parameter to base the skeletal maturation diagnosis [32] due to the high variability of midpalatal suture development stages during a patients’ life [52]. Skeletal effects derived from maxillary expansion have been observed to be greater in young patients, at the prepubertal stages, while pubertal or post pubertal stages can have greater dentoalveolar effects [49]. However, approximately 11% of adult population still present a pubertal stage 4 CVM. This percentage is not high, but it should be considered relevant from a clinical standpoint [53].

Garrett found similar values to our study for alveolar bending after RME with the use of tooth-borne expanders observing a 13% (0.84 mm) of alveolar bone bending, but higher dental tipping effect of 39% at the premolar (2.34 mm) and 49% (3.27 mm) at the first molar from the total expansion achieved in patients with a mean age of 13.8 years [54]. These data show a trend of decreasing orthopedic skeletal effect, increasing alveolar bending and orthodontic tipping from anterior to posterior in line with previous reports [55,56]. Compared to bone-borne maxillary expanders, tooth-born expanders showed as twice as large alveolar bone bending effect [57].

### 4.1. Skeletal Changes

In our study, a total expansion of 4.5 (1.8) mm was achieved after MARPE, defined as the change between the intermolar width at the level of the first molar (IMW). This change was slightly lower compared to other report values [34], but in line with other study [33]. This may be due to the fact that no overcorrection was performed, but enough to correct transversal discrepancy. Buccal and palatal maxillary width showed a significant increase in all the teeth, confirming earlier studies’ results. Significant nasal and maxillary width changes were seen, thus explaining a disjunction effect in the nasal cavity as some studies show [13,14,15].

The highest proportion of the enhanced total expansion, corresponds to skeletal expansion (60%), determined by mean midpalatal suture expansion (SEM) measured in the middle of the palate at first molar level (2.7 (1.0) mm), coincident with other studies using the same measurements method and type of expander (2.55 ± 0.71 mm) [34] over skeletally mature patients (CVM 4) [49] similar to our sample. Ngan et al. [34] reports a 41% of skeletal expansion (SE), with a 2.55 ± 0.71 mm of midpalatal suture expansion (SEM), and a total expansion (TE) of 6.26 ± 1.31 mm, which means that proportionally we have enhanced a higher percentage of skeletal expansion considering that most of our patients where categorized as skeletally mature (stage IV, and V) [49] with a mean age of 17.1 ± 3.5 years. MARPE clinical efficacy and stability performed in young subjects (between 19 and 26 years) report success rates of 86.96%, maintaining achieved skeletal and dentoalveolar changes after disjunction, as well as periodontal structures solidity during retention period [31].

Regarding suture opening, the available literature reports a marked suture opening pattern [44] describing a tendency to result in a pyramidal pattern in the vertical aspect. According to some studies, the triangular suture opening pattern presents the apex in the nasal area and the base in the dental cusps, regardless of the mechanism used [26,37,58]. Lim et al. support MARPE is not only limited to the maxilla, involving also circummaxillary structures [25]. Studies using tooth-borne expanders tend to describe a triangular pattern on the sagittal view for suture opening, wider at the base of anterior maxillar portion [54,57,59] whereas a more parallel pattern was found for bone-borne expanders [57]. These findings could also could be influenced by the location of the appliance [34]. Expanders are tended to be placed in the intermediate palatal position, described as “palatal T zone” [36] in order to assure a higher bicortical anchoring and a more parallel suture opening pattern [60]. From our results, we could describe a uniform almost parallel opening along the mid palatal suture in the axial view, not observing differences superior to 0.4 mm as we move to the molar area, consistent with previous studies using bone-borne or tooth bone-borne expanders [30,34,57,61].

The midpalatal suture expansion, on the coronal cross-sectional slice through the midportion of first molar, evaluated at the Nasal and Palatal Floor levels, showed no statistically significant differences between them, with a mean of 2.4 (1.0) mm, confirming a parallel opening suture pattern also on the coronal view. These values are in congruence with the dimension of suture expansion (SEM) at the first molar level on the axial view with a mean of 2.7 (1.0) mm.

### 4.2. Dentoalveolar Changes

A smaller proportion (40%) corresponds to the dentoalveolar component (1.8 mm). Alveolar bone bending (ABB) and dental inclination (DI) showed reduced mean values of 0.7 (1.6) mm and 1.1 (1.3) mm, respectively. Flexion of the alveolar bone accounted for 15.5% of the total expansion and 24.4% of the dental inclination corresponding to the first molar. Ngan [34] reported a higher dentoalveolar effect of 59%, observing 12% of alveolar bone bending and 47% of first molar dental tipping (2.98 ± 0.56 mm). These results are in agreement with Choi [31] who reported an 87% success for orthopedic expansion in an adolescent sample, where 43% of TE was derived from skeletal expansion [31].

Recent studies evaluating RME effect on periodontal structures found significant reductions in buccal bone thickness (BBT) [29,54], while others reports did not find any or only found minimal changes [62,63]. Garib [20] observed a buccal bone thickness (BBT) reduction from 0.6 to 0.9 mm after RME with no statistical differences between tooth tissue and tooth-borne expanders, regarding the buccal movement of the posterior teeth [20]. A retrospective study treated with MARPE with tooth bone-borne expanders [61] described a maxillary transverse deficiency correction reporting, 37.0% of skeletal expansion, 22.2% of alveolar expansion and 40.7% of dental expansion, observing buccal bone thickness (BBT) (0.6–1.1 mm) and buccal alveolar crest Level (BACL) (1.7–2.2 mm) reductions. Despite the decrease in thickness and height of the buccal bone accompanied with buccal tipping of the maxillary first molar, a high percentage of skeletal expansion was achieved (37%). Some of these changes are comparable to those observed after conventional RME [20,38,64].

Significant changes were found in our study regarding buccal bone thickness (BBT) reductions which ranged from 0.1 (0.2) to 0.4 (0.4) mm for the premolar and molars, but no significant changes were observed after treatment at any site with regards to buccal alveolar crest Height BACL. Ngan [34] reported similar slight changes with a mean buccal bone thickness reduction from 0.27 mm to 0.60 mm at the first molar where bands were placed. As for palatal bone thickness (PBT), there were significant increases from 0.1 to 0.2 mm on the left side for the three teeth, and a small buccal bone thickness (BBT) reduction <0.5 mm was observed in same location after maxillary expansion. Therefore we can deduce that these changes tended to show a more skeletal maxillar expansion pattern, coincident with other studies reporting bilateral palatal bone thickness augmentation after MARPE [29,65] or after RME [20,38]. Toklu et al. [29] found an equivalent reduction in buccal bone thickness, but a palatal bone thickness increase in the anchored teeth with the use of tooth borne and tooth bone-borne expanders after RME, observing a buccal bone thickness reduction in the banded first molars (approximately 0.7–1.2 mm) with both types of expanders in patients with a mean age of 13.8 years.

Dentoalveolar changes such as reduction in buccal bone thickness (BBT) and augmentation of alveolar bone height or vertical buccal alveolar crest Level (BACL) have been confirmed to be greater with the use of tooth-born expanders by one recent prospective comparative study on 60 adolescent patients treated with MARPE tooth bone-borne maxillary expanders or tooth-born expanders [66].

### 4.3. Dental Changes

Buccal teeth inclination and dentoalveolar structures flexion are common findings after RME. However, studies report small statistical relevance. When comparing dental effects related to the use of tooth and tooth bone-borne expanders, one study observed differences which are not statistically significant in terms of absolute dental tipping between groups for the first premolar when this was banded, finding a dental inclination increase of 2.33° (3.03)° with a tooth-borne expander, whereas it remained unchanged in the tooth bone borne expander group (not banded) [29]. However, this finding should be cautiously interpreted due the great individual variability that has been observed for tipping as shown by previous studies [38,67].

In our study, dental inclination measured in degrees did not show any significant variation after treatment, being minimal on all the evaluated teeth, showing a tendency to positive tipping, slightly increasing from the first molar to the first premolar. Therefore, it could be said that MARPE with tooth bone-borne expanders is an effective and safe treatment to correct maxillary transversal discrepancy in adolescent patients when the midpalatal suture is still not fully ossified and partially open.

Some authors state that a 1–24° molar inclination increase is inevitable, probably due to alveolar bending and posterior teeth tipping effect [61,68]. A previous study evaluating this effect after RME observed a significant bilateral first molar buccal tipping with mean values of 5.6°−6.2° [51]. Park et al. [61] reported similar values after MARPE, observing a higher degree of buccal tipping in the first molar compared to the first premolar, related to the buccal bone thickness (BBT) and the crest height (BACL) reduction observed in their study. Perhaps these results can be influenced by a greater mean age of this sample (20.1 ± 2.4 years, range: 16–26 years) compared to our sample. In line with these results, other research reported similar inclination changes, but with lower tipping values of 2.5° [20], and 3.9°, respectively [69]. On the other hand, one recent study found greater first molar inclinations between 4.95°−6.99° after MARPE, but such differences were not significant when compared to first premolar inclinations [61]. Lim et al. [25]. observed a reduction of 0.23° per year for buccal tooth inclination after MARPE completion, which is associated to the previously explained remodeling of buccal bone apposition. In this same line of research, Lagraverè et al. [12] in a comparative study between tooth-borne and tooth bone-borne expander with control group, showed similar changes in the transverse dimension and a significant crown inclination in the posterior segments with both type of appliances.

The high potential of CBCT for evaluating maxillary structures has been confirmed. Good resolution, accuracy (only about 2% magnification), precision, non-invasiveness, lower effective radiation dose compared to other diagnostic methods, with shorter acquisition times have been described as its main advantages (60 s) [70,71,72,73].

Despite all efforts to minimize patient radiation exposure as much as possible, an optimal quality image is needed to perform accurate measurements. Voxel size influences the final image, thus affecting the accuracy of the performed measurements [74,75]. Several studies assessing linear measurements in skull and jaw bones in CBCT have been carried out [76,77,78]. Unfavorable effects such as poor image sharpness and artifacts derived from CBCT imaging are inevitable and can influence alveolar accuracy measurements [74,79]. Specifically related to alveolar bone dimensions, the study of Sun et al. concluded that alveolar bone-height and thickness measurements can be achieved with CBCT images with good to excellent repeatability, pointing out that decreasing voxel size from 0.4 to 0.25 mm can improve alveolar bone linear measurement accuracy, observing that measures with a voxel size of 0.25 mm were closer to the direct measurements than when using 0.4 mm [74].

However, another study evaluating bone height and width, showed that 0.4 mm voxel images provided results as accurate as 0.125 mm voxel images [80]. Moreover, Torres and coauthors did not find differences between voxel sizes of 0.2, 0.3 and 0.4 mm, when evaluating linear bone measurements, in agreement with previous results [81]. Although a higher radiation dose when using lower voxel size values is inevitable, this can be justified by its higher resolution needed for this type of measures. A 0.2 voxel size was used in our study in accordance with other similar research considered as reference [29,57].

Some limitations such as small sample size and the short-term follow up have been considered and discussed, though the desired goals have been fulfilled. Moreover, a control group could be used to compare different expander designs or treatments. However, no sufficient homogeneous sample could be collected. On the other hand, the high precision of quantitative analyses on CBCT images contributes to the reliability of the outcomes, making the small sample size acceptable [20]. Therefore, it would be interesting to confirm our results in future studies with longer retention periods and larger samples.

## 5. Conclusions

From the analysis of our results, we can conclude that:

1. MARPE with tooth bone-borne expanders is an effective method for treating maxillary deficiency in adolescent patients with incomplete ossification of the midpalatal suture, observing a significant maxillary expansion.
2. Although, some periodontal or dentoalveolar changes such as buccal bone thickness reduction were observed radiographically after treatment, none of these effects were clinically detectable.
3. Maxillary deficiency correction with MARPE resulted in a larger skeletal expansion, with reduced dentoalveolar and dental effects and the buccal alveolar crest remained practically unchanged.
4. Tendency of parallel midpalatal suture opening pattern was observed after treatment in the coronal and axial view.
5. Aside from the use of tooth bone support for maxillary expansion, no significant dental inclination effect was observed after treatment.

## Figures and Tables

**Table 1:** Sample age and maxillary compression distribution. Appliance’s activation time. Age (years), Maxillar compression (mm), Appliance activation time (days).

	N	Min	Max	Mean Difference (SD)	IC 95% Mean	Median	Median (P_25_;P_75_)	IC95% Median
Age	15	13.0	24.0	17.0 (4.0)	15.0;19.0	16.0	(14.0;20.0)	16.0; 21.0
Maxillary compression	15	2.5	8.7	5.4 (2.1)	4.2; 6.5	5.1	(3.7;7.5)	3.8; 7.5
Appliance activation time	15	10.0	35.0	22.0 (8.0)	17.0; 26.0	20.0	(15.0;30.0)	15.0; 30.0

**Table 2:** Description of radiographic CBCT measurements performed with In vivo software program. Standardized coronal and axial sections for radiographic measurements of first and second premolars, and first molars.

Nasal width (NW)	Distance between right and left most lateral point of the nasal cavity in the coronal section at the level of first molar where total length of palatal root and crown are visualized.	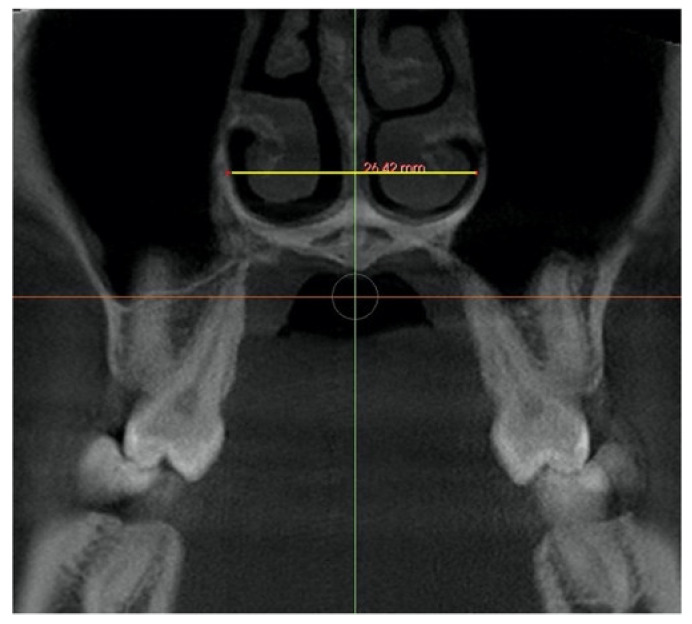
Maxillary width (MW)	Distance between the lowest point of lateral right and left contour concavities of the maxillary bone, on a coronal section, at the level of first molar where total length of palatal root and crown are visualized.	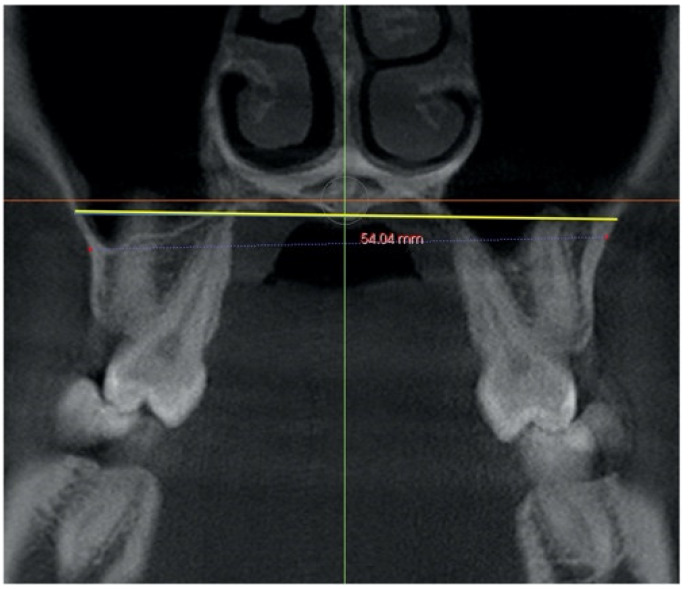
Palatal suture opening (SO)	Distance between the external right and left maxilla edges in the axial view generating a slice in the horizontal plane, allowing a good visualization of the midpalatal suture at first and second premolar, and first molar. The edges were identified with a small point on an axial cross-sectional slice at the level of the first molar trifurcation.	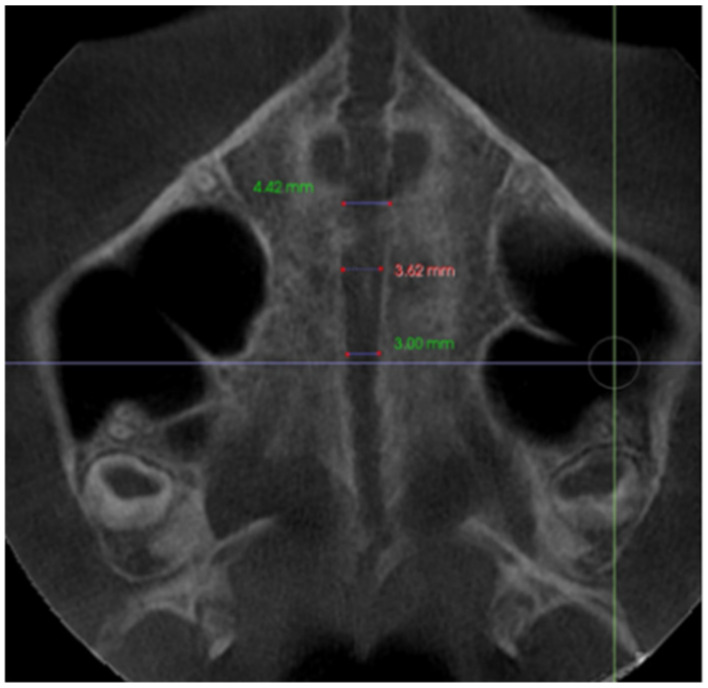
Buccal maxillary width (BMW)	Distance between right and left most prominent point of the buccal bone crest in first and second premolar, and first molar, in the first anterior coronal cut described for each tooth.	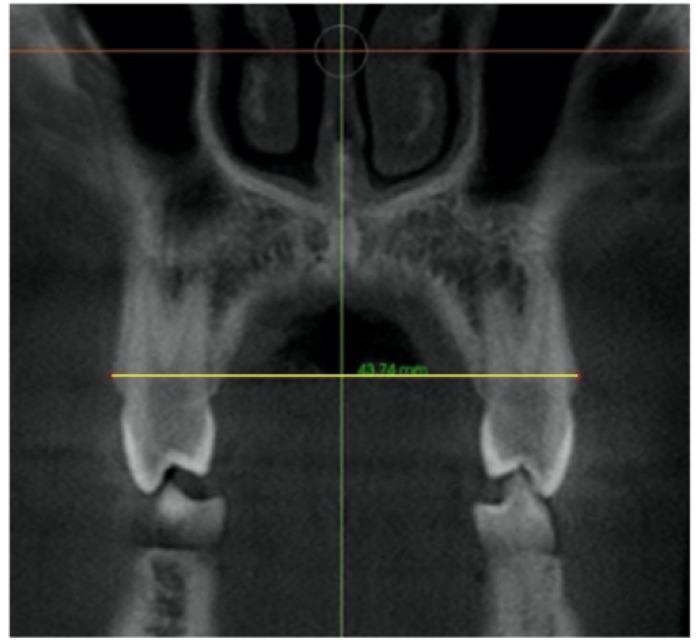
Palatal maxillary width (PMW)	Distance between right and left most prominent point of palatal bone crest in first and second premolar, and first molar, in the first anterior coronal slice as describe for each tooth. At the level of the buccal maxillary width.	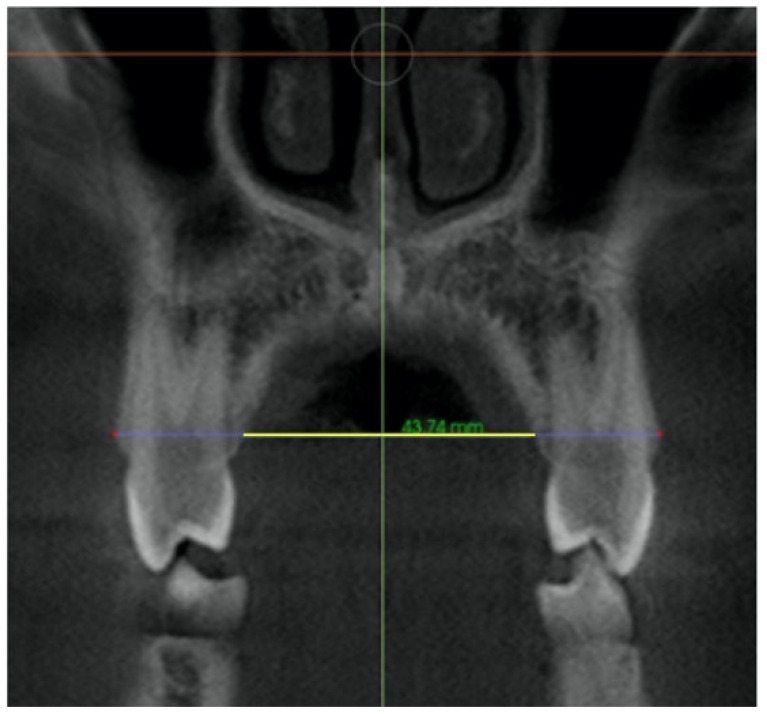
Buccal bone thickness (BBT)	Distance from the external border of the buccal cortical plate to the center of buccal aspect of first and second premolar root, and from the external border of buccal cortical plate to the center of the messiobuccal and distobuccal root of first molar, in an axial section parallel to the palatal plane, at the level of the first molar right (R) and left (L) trifurcation for each side.	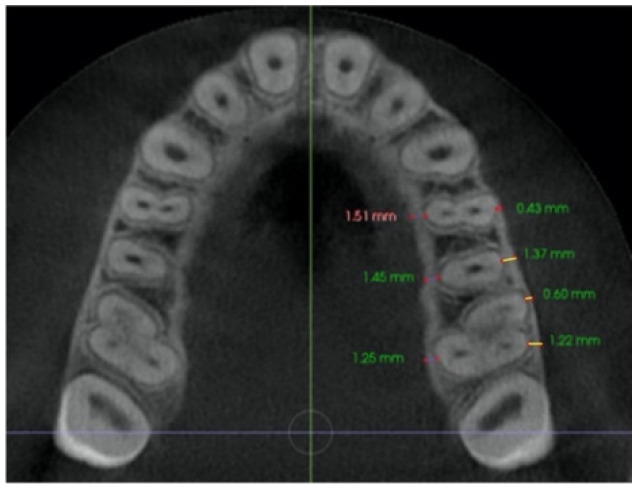 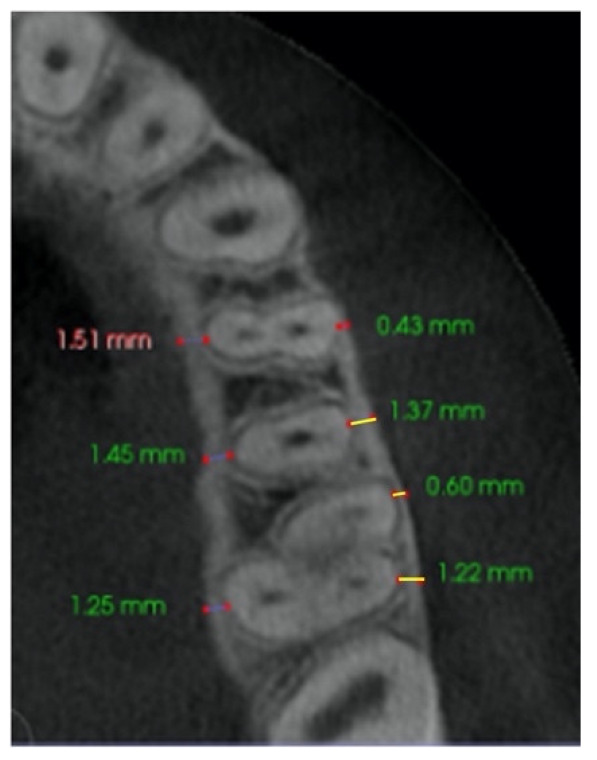
Palatal bone thickness (PBT)	Distance from the external border of palatal cortical plate to the center of palatal aspect of first and second premolar root, and first molar palatal root, in an axial section parallel to palatal plane, at the level of the first molar right (R) and left (L) trifurcation for each side.	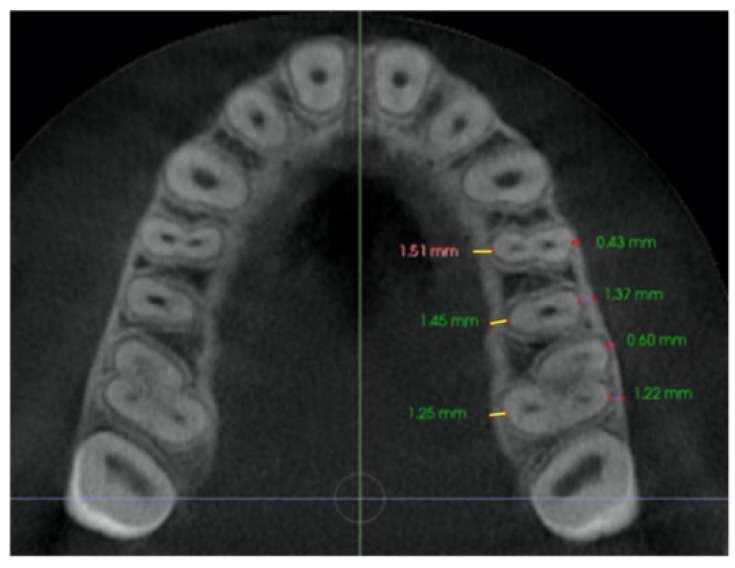 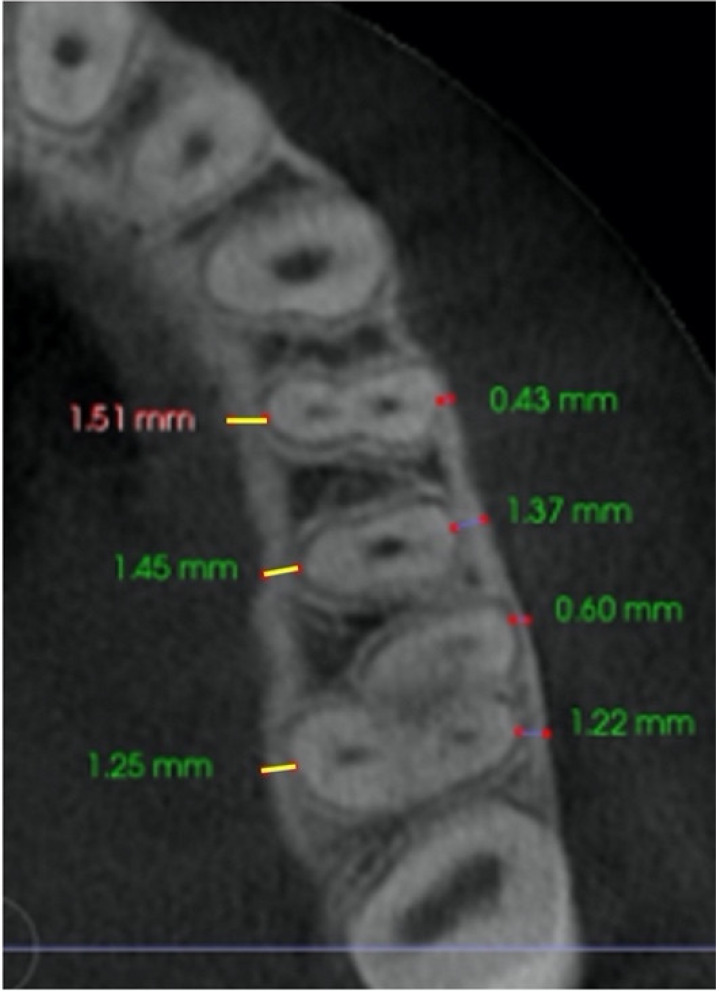
Buccal alveolar bone crest level (BACL)	Distance from the tip of buccal cusp to the buccal bone crest of first and second premolar, and from the mesiobuccal cusp of first molar crown, to the buccal bone crest in the first anterior coronal cut describe for each tooth, on the right (R) and left (L) side.	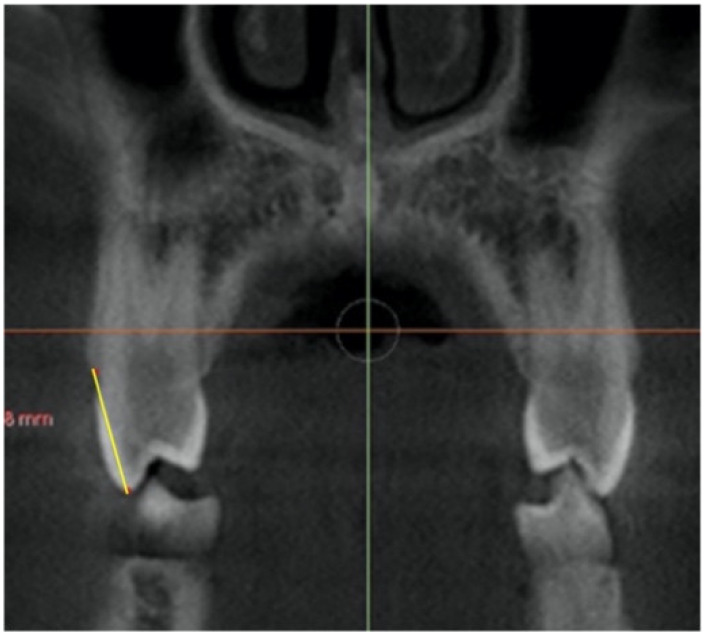
Dental inclination (INCL)	Angle formed by the intersection of two tangents that passed through the buccal and palatal cusps of the first and second premolars of both contralateral teeth, and through the messiobuccal and palatal cusp of the first molar, in the coronal section.	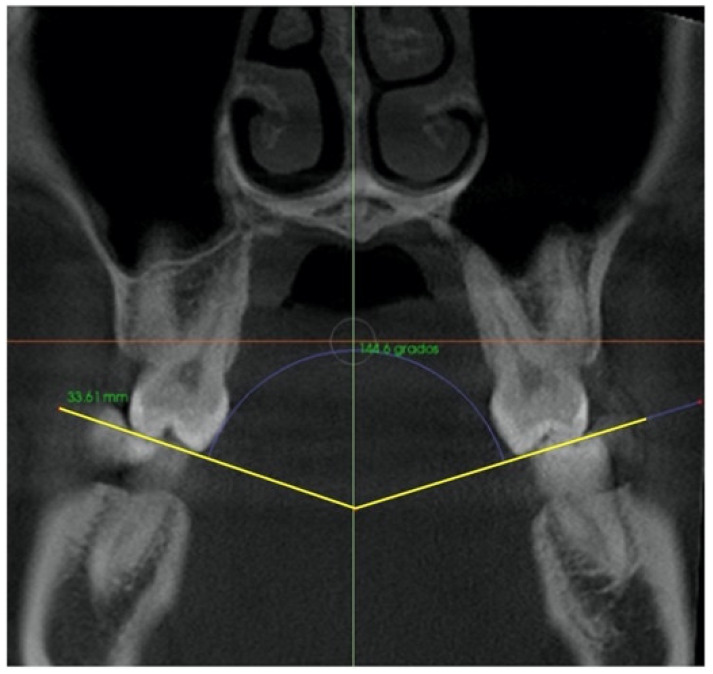

**Table 3:** Evaluation of the suture opening pattern in the coronal section and midpalatal suture expansion at the nasal and palatal floor levels.

Nasal Floor (NF)	Distance from right and left external edges of the palatine suture at the level of the nasal floor in the coronal view. On a coronal cross-sectional slice through the center of the first molar. The suture external edges were verified in the axial cross-sectional slice.	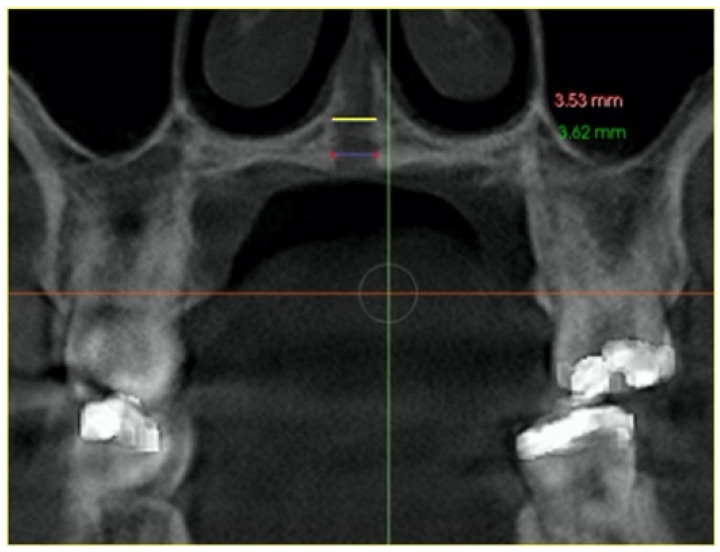
Palatal Floor (PF)	Distance from right and left external edges of the palatine suture at the level of the palatal floor. On a coronal cross-sectional slice through the center of the first molar. The suture external edges were verified in the axial cross-sectional slice.	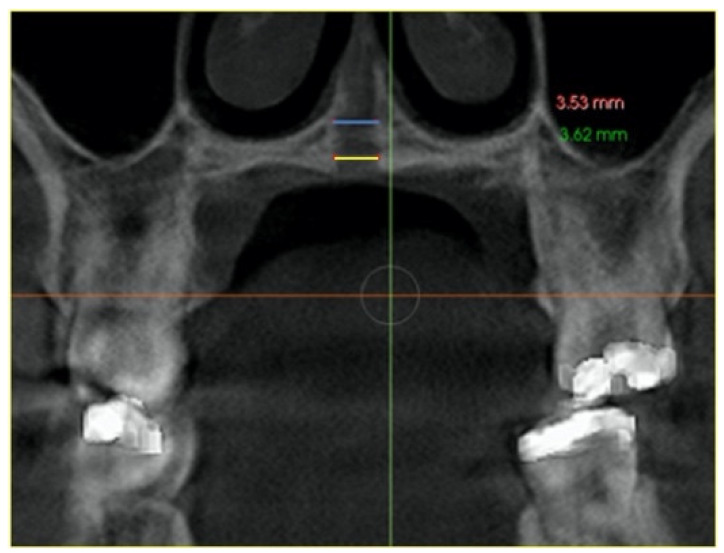

**Table 4:** Radiographic CBCT measurements to quantify skeletal, dentoalveolar and dental expansion. Sutural expansion, intermolar width and palatal maxillary width (SEM, IMW and PMW) were measured at first molar and quantified on the same coronal cross-sectional slice.

Sutural expansion (SEM)	The sutural expansion in the middle of the palate. Distance between the left and right external border of the palatal aspect of the maxilla, in the middle of the palate between the palatine bone and the nasal floor, on a coronal cross-sectional slice at first molar. Through the midportion of the first molar.	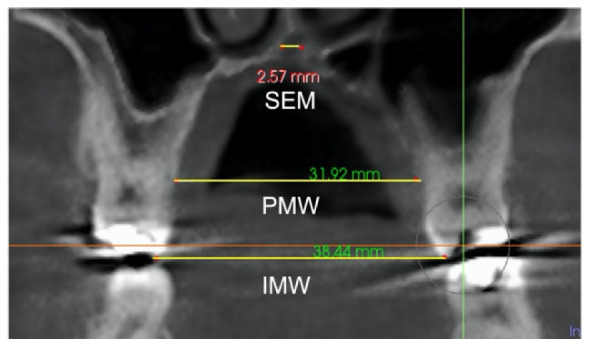
Intermolar width (IMW)	Distance between right and left tip of palatal cusp of first molars in a coronal cross-sectional slice through the midportion of first molar.	
Palatal maxillary width (PMW)	Distance between the right and left external border of the palatal maxillary bone, on a coronal cross-sectional slice at first molar. Through the midportion of the first molar.	

**Table 5:** Sample sex, cross bite, skeletal maturation and palatal sutural stage distribution.

		N Number (%)	95% CI
Sex	Male	3 (20)	6.0; 44.4
	Female	12 (80)	55.6; 94.0
Cross Bite	Absence	3 (20)	6.0; 44.4
	Bilateral	12 (80)	56.6; 94.0
Skeletal maturation	Stage I	0 (0)	0.0; 21.8
	Stage II	0 (0)	0.0; 21.8
	Stage III	2 (13.3)	2.9; 36.3
	Stage IV	7 (46.7)	23.9; 70.6
	Stage V	6 (40.0)	18.8; 64.7
	Stage VI	0 (0)	0.0; 21.8
Palatal Sutural Stage	Stage A	0 (0)	0.0; 21.8
	Stage B	1 (6.7)	0.7; 27.2
	Stage C	13 (86.7)	63.7; 97.1
	Stage D	1 (6.7)	0.7; 27.2
	Stage E	0 (0)	0.0;21.8

**Table 6:** Comparison of Pre and Posttreatment Skeletal measurements. Skeletal changes comparing CbcT1-CbcT2 measurements. BMW: Buccal maxillary width. PMW: Palatal maxillary width. NW: Nasal width. MW: Maxillary width. SO: suture opening. 1PM: first premolar; 2PM: second premolar and M1: first molar.

T1–T2	N	Min	Max	Mean Difference (SD) mm	IC95% Mean	Median (mm)	Median (P_25_; P_75_)	IC95% Median	*p*-Value
NW	15	0.4	4.6	2.1 (1.1)	1.5; 2.7	2.3	1.1; 2.7	1.3; 2.7	0.00005
MW	15	0.4	6.2	2.5 (1.6)	1.6; 3.4	2.1	1.3; 3.8	1.9; 3.8	0.00005
SO1PM	15	1.7	6.0	3.3 (1.3)	2.5; 4.0	2.7	2.4; 4.4	2.4; 4.4	0.00005
SO2PM	15	1.2	6.0	2.9 (1.4)	2.1; 3.6	2.4	1.6; 3.8	1.7; 3.8	0.00005
SO1M	15	1.0	6.0	2.6 (1.3)	1.9; 3.3	2.2	1.8; 3.4	1.8; 3.4	0.00005
NF	15	1.1	4.7	2.4 (1.0)	1.9; 3.0	2.4	1.7; 3.1	1.7; 3.1	0.000
PF	15	1.0	4.5	2.4 (1.0)	1.8; 3.0	2.6	1.6; 3.2	1.8; 3.6	0.000
SEM	15	0.7	4.7	2.7 (1.0)	2.1; 3.1	2.6	1.9; 3.3	2.2; 3.3	0.000

**Table 7:** Comparison of Pre and Post treatment Dentoalveolar measurements. Dentoalveolar changes comparing CbctT1-CbctT2 measurements. BBT: buccal bone thickness. MB: messiobuccal root of first molar. DB: distobuccal root of first molar. R: right side. L: left side. PBT: Ppalatal bone thickness. BACL: bone alveolar crest level. 1PM: first premolar; 2PM: second premolar and M1: first molar.

T1–T2	N	Min	Max	Mean difference (SD) mm	IC95% Mean	Median (mm)	Median (P_25_; P_75_)	IC95% Median	*p*-Value
BMW1PM	15	0.6	7.8	3.7 (2.0)	2.6; 4.8	3.5	2.1; 5.0	2.2; 5.0	0.00005
BMW2PM	15	0.8	14.8	4.1 (3.6)	2.1; 6.1	2.6	2.2; 4.3	2.4; 4.3	0.001
BMW1M	15	1.1	9.7	3.7 (2.1)	2.6; 4.9	3.3	2.6; 4.0	3.1; 4.0	0.001
PMW1PM	15	1.0	8.4	3.7 (1.8)	2.7; 4.7	3.4	2.6; 4.8	2.9; 4.8	0.00005
PMW2PM	15	1.6	7.8	3.2 (1.6)	2.3; 4.1	3.0	2.2; 3.6	2.8; 4.4	0.001
PMW1M	15	−5.6	7.2	2.2 (2.6)	0.7; 3.6	2.3	1.7; 3.0	1.8; 3.0	0.009
BBTR1PM	15	−1.0	0.0	−0.3 (0.3)	−0.4; −0.1	−0.2	−0.4; 0.0	−0.2; −0.0	0.003
BBTR2PM	15	−0.4	0.3	−0.1 (0.2)	−0.2; −0.0	−0.2	−0.2; −0.0	−0.2; −0.0	0.013
MBBTR1M	15	−0.9	0.0	−0.3 (0.3)	−0.5; −0.2	−0.2	−0.6; −0.1	−0.3; −0.1	0.001
DBBTR1M	15	−0.9	0.0	−0.3 (0.3)	−0.5; −0.2	−0.3	−0.5; −0.2	−0.4; −0.2	0.00005
BBTL1PM	15	−1.3	−0.2	−0.3 (0.4)	−0.6; −0.1	−0.2	−0.5; −0.1	−0.5; −0.1	0.004
BBTL2PM	15	−0.6	0.0	−0.3 (0.2)	−0.3; −0.1	−0.3	−0.4; −0.1	−0.3; −0.1	0.00005
MBBTL1M	15	−1.1	0.0	−0.4 (0.4)	−0.6; −0.2	−0.3	−0.7; 0.0	−0.6; 0.0	0.003
DBBTL1M	15	−1.5	0.0	−0.4 (0.4)	−0.6; −0.2	−0.3	−0.7; −0.1	−0.5; −0.1	0.001
PBTR1PM	15	−1.8	1.1	0.1 (0.7)	−0.3; 0.5	0.2	−0.3; −0.4	−0.0; 0.4	0.552
PBTR2PM	15	−0.7	0.8	0.2 (0.4)	−0.4; 0.5	0.3	0.0; 0.4	0.0; 0.4	0.09
PBTR1M	15	−0.2	0.9	0.1 (0.3)	−0.0; 0.3	0.0	0.0; 0.3	0.0; 0.3	0.073
PBTL1PM	15	−0.4	0.6	0.2 (0.2)	0.0; 0.3	0.2	0.1; 0.3	0.1; 0.3	0.013
PBTL2PM	15	−0.3	0.5	0.1 (0.2)	0.0; 0.3	0.2	0.1; 0.3	0.1; 0.3	0.012
PBTL1M	15	−0.1	0.5	0.2 (0.2)	0.1; 0.3	0.1	0.0; 0.4	0.1; 0.4	0.003
BACLR1PM	15	−2.3	0.7	−0.1 (0.7)	−0.5; 0.3	0.0	−0.2; 0.2	0.0; 0.3	0.448
BACLR2PM	15	−1.1	0.5	−0.1 (0.5)	−0.4; 0.2	0.0	−0.4; 0.2	−0.1; 0.2	0.420
BACLR1M	15	−2.2	1.0	−0.0 (0.8)	−0.5; 0.5	0.2	−0.6; 0.6	−0.2; 0.6	0.493
BACLL1PM	15	−1.2	0.3	−0.2 (0.4)	−0.4; 0.0	0.0	−0.2; 0.1	−0.0; 0.1	0.012
BACLL2PM	15	−1.6	0.7	−0.2 (0.5)	−0.5; 0.1	−0.1	−0.6; 0.1	−0.5; 0.1	0.145
BACLL1M	15	−0.4	1.5	0.2(0.6)	−0.1; 0.5	0.1	−0.1; 0.4	−0.1; 0.4	0.144

**Table 8:** Comparison of Pre and Post treatment Dental inclination measurements. Dental inclination changes after maxillary expansion comparing CbctT1-CbctT2 measurements. INCL: inclination. 1PM: first premolar; 2PM: second premolar and M1: first molar.

T1–T2	N	Min	Max	Mean Difference (SD) mm	IC 95% Mean	Median mm	Median (P_25_;P_75_)	IC95% Median	*p*-Value
INCL1PM	15	−33.6	22.4	−1.6 (15.9)	−10.4; 7.3	−0.2	−13.2; 8.6	−2.0; 8.6	0.71
INCL2PM	15	−22.5	32.3	−2.0 (15.6)	−10.6; 6.7	−5.7	−14.0; 6.0	−12.9; 6.0	0.62
INCL1M	15	−15.3	21.7	−3.3 (9.4)	−8.5; 1.9	−3.7	−10.0; −1.3	−8.0; −1.3	0.200
